# Comprehensive Analysis of Formin Genes Reveals Their Roles in Tissue Development and Cold Stress Responses in *Brassica rapa*

**DOI:** 10.3390/genes17020207

**Published:** 2026-02-09

**Authors:** Nan Wang, Shangjia Liu, Bingxue Han, Zekun Hu, GuangYao Chen, Yanhua Wang, Gengxing Song, Yinqing Yang

**Affiliations:** 1 College of Horticulture Science and Engineering, Shandong Agricultural University, Tai’an 271000, China; 2 Agricultural College, Hengxing University, Qingdao 266000, China; 3 Shandong Hualiang Seed Co., Ltd., Weifang 261000, China

**Keywords:** *Brassica rapa*, formin homology, tissue development, cold stress

## Abstract

Background: Formin proteins are crucial regulators of actin filament assembly and elongation in eukaryotic cells, playing important roles in plant development and abiotic stress responses. However, the functional characterization of formins in *Brassica rapa* L. remains undiscovered. Methods: A total of 27 formin family members (*BrFH*s) were identified through genome-wide alignment with *Arabidopsis thaliana* (L.) Heynh. Results: Phylogenetic analysis classified *BrFH* gene family into two distinct clades, designated Group I and Group II, which exhibit divergent protein architectures. Promoter analysis revealed that *BrFH*s contain multiple cis-regulatory elements related to growth and development, stress responses, and phytohormone signaling. These findings suggest that *BrFH*s may have diversified functions. Tissue-specific expression analysis revealed that *BrFH*s exhibit distinct expression patterns across various tissues. Notably, *BrFH15* and *BrFH18* are highly expressed in flowers, displaying expression profiles similar to those of floral development genes such as *AP3*, *AGL10* and so on. Additionally, many *BrFH*s show dynamic expression patterns in response to cold stresses. In particular, *BrFH2*, *BrFH19* and *BrFH27* were up-regulated, and their co-expression within the gene network suggests potential roles in regulating cold stress. Conclusions: These results clarify the functional roles of *BrFH*s and shed light on the molecular mechanisms underlying their regulation of tissue development and responses to cold stress in *Brassica rapa*.

## 1. Introduction

The cytoskeleton in eukaryotes plays a various role in cell division and differentiation, growth and development, signal perception, and cellular immunity, which is mainly composed of two highly conserved compounds, microfilaments and microtubules. The coordinated reconstruction and dynamic behavior of these structures are regulated by various actin binding proteins (ABPs) [1,2,3,4]. In plant species, ABPs mainly include six kinds, among which formin proteins have been well characterized as actin binding proteins, defined as nucleation factors that initiate microfilament polymerization and then participated in the structural organization of actin cytoskeleton. Plant morphology is relatively conserved in the kingdom plantae, representatively defined by the conserved FH2 domain [5,6,7,8,9]. In addition, a polygenetic analysis based on multiple sequence alignment showed that the formin homologs are not only present in plant genomes, but also in fungi and metazoans, indicating the universality of the formin homology protein family in eukaryotes [10]. Additionally, researchers have extensively reported that the organization and dynamics of the cytoskeleton can be altered in response to external stress stimuli to regulate plant growth and development, mainly depending on the action of formin homology proteins [11,12].

Over the past decades, formin homology proteins have extensively attracted much research attention in numerous physiological and cellular processes, especially for cell morphology, signal transduction, and stress adaptation [13,14]. Several plant species have identified formin homology gene family. For example, 21-formin encoding proteins have been found in *Arabidopsis* (*Arabidopsis thaliana*) [15], 17 proteins in rice (*Oryza sativa*) [16] and 46 proteins in cotton (*Gossypium hirsutum*) [17]. The model plant *Arabidopsis* genome have been described, which have been grouped into two distinct subfamilies based on the sequence similarity and domain composition, Class I and Class II [11,18]. *AtFHs* have been universally reported to be involved in numerous physiological processes and responses to external stimuli, such as drought stress, salt stress, and other abiotic stresses [19,20]. For example, the *Arabidopsis* plants expressing formin *AtFH1* had the function of regulating actin filament bundles, and mainly participated in pollen tube broadening and growth depolarization [15,21]. In addition, cold stress can induce the expression of FH gene, which can promote the recombination of cytoskeleton to cope with the risk of membrane lipid coagulation, thereby ultimately affecting on the polar growth of pollen tubes, which underscored their vital role in controlling the normal growth and development of pollen tubes by regulating actin polymerization [22]. Similarly, high temperature stress condition not only had a significant impact on the thermal stability of microfilament proteins, but also altered the polar transport of pollen tubes, and even led to pollen abortion [23]. The actin-depolymerizing factors (ADFs) have been functionally characterized as a class of conserved microfilament-binding proteins to regulate the rapid turnover of the microfilament cytoskeleton, rearrange construction and finally contribute to proper pollen tubes growth or root hairs growth. The formin *AtFH4* regulated the lateral intercellular boundaries of the plant root bark and cortex, which plays a role in linking the membrane with the cytoskeletal network [24], but under the external stress, the expression of *AtFH4* showed a suppression, and ultimately affected the formation of microtubules and actin-associated proteins [11]. Moreover, *AtFH6*, a member of *Arabidopsis* formin, was reported to impact the nucleation of the barbed end of actin filaments and promoted root hair cell, while the mutant of *AtFH6* showed an abnormal development during the mineral salt deficiency [19]. The conserved domain of *AtFH8* regulates the nucleation of the barbed end of actin filaments and promotes root hair cell and polarized cell growth [25]. Class II members were accompanied by a N-terminus phosphatase and tensin-related (PTEN)-like domain, which intricately connected the cell with external environments [26]. Notably, *Arabidopsis FH12*, classified as a Class II formin, was reported to positively regulate the normal dynamics of actin filaments in root hairs under salt stress, serving as evidence that mutants lacking FH12 pronounced inhibition of root elongation [27]. In addition, *AtFH14* and *AtFH16* have also been reported to be able to promote taproot growth and increase the number of lateral roots [28,29]. In other plant species, the *MtFH* genes were significantly up-regulated under drought and salt stress, which may reduce water loss or ion toxicity by enhancing the stability of the cytoskeleton in *Medicago truncatula* [30]. The *GhFH* genes played significant roles in development of cotton (*Gossypium hirsutum* L.) under heat and salt stresses [31]. Gene expression identified *FH* genes with a dynamic pattern in response to salt and drought stresses in soybean (*Glycine max* L.), which revealed the potential roles in regulating plant stress adaptation [32]. Meanwhile, there is a synergistic effect between *FH* gene and stress signaling pathway, for example, the closure of stomata was regulated by signaling molecules under drought stress, including abscisic acid (ABA), reactive oxygen species (ROS), and phospholipids. Among them, actin filaments in the guard cells underwent recombination and played a role in stomatal movement [33]. Consequently, the dynamic adjustment of the cytoskeleton is crucial for plants to adapt to environmental changes under abiotic stresses.

*Brassica rapa* (*B. rapa*) is one of the most extensively cultivated vegetables, and represents one of the major vegetables in China. To cope with these unfavorable growth conditions caused by stresses (e.g., cold stress), Chinese cabbage has evolved complex regulatory mechanisms, particularly during vegetative growth stages, enabling its survival and maximizing its achievable yield and quality [34,35]. Concurrently, it has been observed that the expression of many genes that respond to cold stress changed due to physiological and metabolic alterations. However, relatively little research has been conducted to date on the function of the formin gene family and its members in other plant species. Therefore, further systematic identification and classification of the formin gene family members in *B. rapa* are necessary to be conducted. In the present study, we performed a genome-wide identification of the *BrFH* gene family, revealing a potential regulatory role of *BrFH* in response to cold conditions to orchestrate plant growth and development. The finding from our study on the *BrFH* gene family not only deepens our understanding of its physicochemical properties and structure, but also leverages their stress resistance function to provide new targets for crop stress resistance breeding.

## 2. Materials and Methods

### 2.1. Identification of FH Genes in the B. rapa Genome and Subcellular Localization Analysis

To identify *BrFH* genes in the *B. rapa* genome, the full-length protein sequences of *FH* genes from *A. thaliana* were retrieved from the TAIR database (https://www.arabidopsis.org/) (accessed on 3 November 2025). These sequences were used as queries in BLAST (v2.15.0) searches against the Plant Information Database to identify homologous sequences in *B. rapa*. Protein domain structures were analyzed using the Pfam database (http://pfam.xfam.org/) (accessed on 12 November 2025) and the SMART tool (v9.0, http://smart.embl-heidelberg.de/) (accessed on 23 November 2025) to construct a genome-wide protein domain model for *B. rapa* [36,37].

The physicochemical properties of the identified *BrFH* proteins were predicted using the Expasy ProtParam tool (http://web.expasy.org/) (accessed on 1 December 2025). Subcellular localization of the proteins was predicted using WoLF PSORT (http://wolfpsort.org/) (accessed on 9 December 2025) [38].

### 2.2. Chromosomal Localization, Synteny, and Phylogenetic Analysis

The chromosomal distribution of *BrFH* family was mapped through genome-wide chromosomal mapping of *B. rapa*, and their locations were visualized using TBtools (v1) [39]. Syntenic relationships of *FH* family between *B. rapa* and *A. thaliana* were analyzed using MCScanX (v1.0.0) to assess evolutionary conservation and collinearity [40].

Phylogenetic analysis was conducted using the maximum likelihood method in MEGA (vX) with 500 bootstrap replicates, while all other parameters were set to default values [41]. The resulting phylogenetic trees were visualized using iTOL (v7) [42].

### 2.3. Motif and Conserved Domain, and Protein Structure Analysis

Conserved motifs were identified using the online software the MEME suite (v5.5.9) [43]. In MEME, the maximum number of motifs was set to 10, and the occurrences of a single motif were set to zero or one per sequence. NCBI’s Batch CD-Search (https://www.ncbi.nlm.nih.gov) (accessed on 18 December 2025) was used to identify conserved domains, and these were visualized using “Visualize Pfam Domain Pattern” (from Pfam Search) in TBtools (v1) [44]. The tertiary structures of *BrFH2* were predicted using AlphaFold (v3) [45], and structural visualization and comparison were performed using PyMOL (v2.6) [46].

### 2.4. Cis-Acting Element Analysis

To investigate the potential regulatory mechanisms of *BrFH* family genes, a 2 kb sequence upstream of the translation start site of each gene was retrieved and subjected to cis-acting regulatory element analysis using the PlantCARE database with default parameters [47]. The type and abundance of these elements were visualized using R (v4.3.3) packages, with heatmaps and bar plots providing an intuitive overview of element types and frequencies across different gene promoters.

### 2.5. Expression Profiling of BrFH Genes in Different Tissues and Under Cold Stress

In order to investigate the expression patterns of *BrFH*s, transcriptomic data from various tissues of *B. rapa*—including roots, stems, leaves, and flowers—were obtained from the NCBI database and analyzed (Table S1). In addition, gene expression under different temperature conditions was examined in cold-tolerant (Longyou-7) and cold-sensitive (Lenox) *B. rapa* accessions to evaluate the potential involvement of *BrFH*s in cold stress responses (Table S1). Control plants were grown at 22 °C with a 16/8 h light/dark cycle, while cold-treated plants were transferred to 4 °C. The leaf were collected, immediately flash-frozen in liquid nitrogen, and stored at −80 °C for RNA extraction. RNA-seq reads were aligned to the *B. rapa* Chiifu v1.5 reference genome using HISAT2 (v2.2.1) [48], and gene expression levels were quantified with featureCounts (v1.6.4) [49]. Transcript abundance was normalized as transcripts per million (TPM), which was used primarily for visualization and comparison of gene expression patterns across tissues/conditions. The resulting expression profiles were visualized using R (v4.3.3) package, enabling the identification of both tissue-specific and cold-responsive expression patterns of *BrFH* genes.

### 2.6. Co-Expression Network Analysis

To investigate potential functional interactions between *BrFH* genes and other genes, a weighted gene co-expression network analysis (WGCNA) was conducted based on gene expression levels across various tissues and under cold stress conditions [50]. Pearson correlation coefficients were calculated to assess the expression relationships between *BrFH* genes and other transcriptionally active genes across various tissues and conditions. The resulting network was visualized using Cytoscape (v3.8.2) [51], enabling the identification of key *BrFH* genes potentially involved in shared biological pathways or regulatory modules.

### 2.7. Gene Ontology (GO) Enrichment Analysis

To gain insights into the potential biological functions of *BrFH* genes, GO enrichment analysis was performed. The genes co-expressed with *BrFH* family members were subjected to GO annotation and enrichment using the eggNOG-mapper (v5.0) [52], TBtools and clusterProfiler package in R (v4.3.3) [53], under default parameters. Significantly enriched GO terms (adjusted *p* < 0.05) were visualized using bubble charts.

### 2.8. qRT-PCR Analysis

Since the transcriptomic data were obtained from public databases, Chiifu was selected for subsequent analyses to further investigate the expression patterns of the candidate genes.

Seeds of Chiifu were sown in an MS-modified medium (containing vitamins, sucrose, and agar) (PM10121-307) in a plant incubator at a temperature of 24 °C, relative humidity: 67%, light for 16 h, and darkness for 8 h. The different test materials (‘848 Egg White’ Chinese cabbage) were subjected to cold stress (4 °C for 0, 6, 12, and 24 h) at State Key Laboratory of Crop Biology, Shandong Agricultural University (Tai’an, Shandong, China). Following exposure to stress, the materials were rapidly frozen in liquid nitrogen for further experiments. *BrFH* genes were subjected to Paired-end (PE) sequencing using Next-Generation Sequencing (NGS) based on the Illumina HiSeq sequencing platform by BioMarker Technologies (Beijing, China), and three biological replicates were collected for each sample.

Total RNA was extracted using a FastPure^®^ Cell/Tissue Total RNA Isolation Kit V2 (Vazyme Biotech Co., Ltd., Nanjing, China); RNA integrity was assessed by 1% agarose gel electrophoresis, and the RNA concentration and purity were measured using a spectrophotometer (Thermo NanoDrop One, Thermo Fisher Scientific Inc., Wilmington, DE, USA). Primer sequences were obtained from the qPrimerDB-qPCR Primer Database (https://biodb.swu.edu.cn/qprimerdb/) (accessed on 22 December 2025). The primer sequences are shown in Table S2. cDNA was obtained by reverse transcription using HiScript^®^ II Q RT SuperMix with a qPCR sample kit (Novozymes, Tianjin, China); it was then diluted tenfold and stored at −20 °C. qRT-PCR reactions were performed in technical triplicates with a final volume of 20 µL, containing the following components: 2 × SYBR qPCR Master Mix 10.0 μL; upstream and downstream primers (10 μmol∙L^−1^) 0.4 μL each; cDNA template 1.0 μL; and ddH_2_O 8.2 μL. The thermal cycling conditions were as follows: 40 cycles of 95 °C for 30 s; 95 °C for 10 s; and 60 °C for 22 s, followed by 95 °C for 25 s; 60 °C for 60 s; and 95 °C for 7 s. The stability of the internal reference gene, *BraActin 2*, was validated using the geNorm algorithm, confirming its suitability for normalization under the experimental conditions. The 2^-∆∆CT^ method was used to analyze expression levels [54]. To quantitatively assess the concordance between the transcriptomic data and qRT-PCR validation, Pearson correlation coefficients were calculated, which exceeded 0.80 for all tested genes, indicating a high reliability of the RNA-seq expression profiles.

### 2.9. Ka/Ks Analysis

Ka and Ks values of FH gene pairs were calculated to evaluate selective pressure during evolution. Ka and Ks values were estimated using KaKs_Calculator (v2.0) under the YN model [55], and unreliable gene pairs were excluded. Ka/Ks ratios were used to infer selection patterns across plant lineages, from chlorophytina to eudicots.

## 3. Results

### 3.1. Genome-Wide Identification and Characterization of FH Family Genes

To identify potential *FH* genes in *B. rapa*, a comprehensive investigation was performed utilizing *FH* genes from *A. thaliana* as a query against the *B. rapa* genome. The resulting candidate genes were then screened for the presence of the FH domain (PF02181.27), which allowed the selection of genes to be further refined. The presence of 27 *FH* genes (designated *BrFH*s) were identified across the genome-wide level in *B. rapa*. These genes were distributed across eight chromosomes in the *B*. *rapa* genome, namely *BrFH1* to *BrFH27* according to their chromosomal positions (Table 1). The expectant polypeptides encoded by *BrFH* genes exhibited a prominent variation in length, ranging from 196 amino acids (*BrFH20*) to 1627 amino acids (*BrFH3*), with predicted relative molecular weights (RMW) spanning from 22.01 kDa (*BrFH20*) to 175.3 kDa (*BrFH3*). The isoelectric point (pI) values exhibited a spectrum from 4.75 (*BrFH20*) to 9.51 (*BrFH1*). Notably, subcellular localization predictions predominantly placed most BrFH proteins within the plasma membrane, while the remaining seven formin proteins were localized to the cytoplasm, six formin proteins were localized to the chloroplast, four formin proteins were localized to the nucleus, and two formin protein was localized to the vacuole. These findings mutually conduce to a nuanced understanding of the structure and functional attributes of identified *BrFH* genes in *B. rapa*.

### 3.2. Phylogenetic Analysis and Classification of FH Family Genes

**Figure 1:**
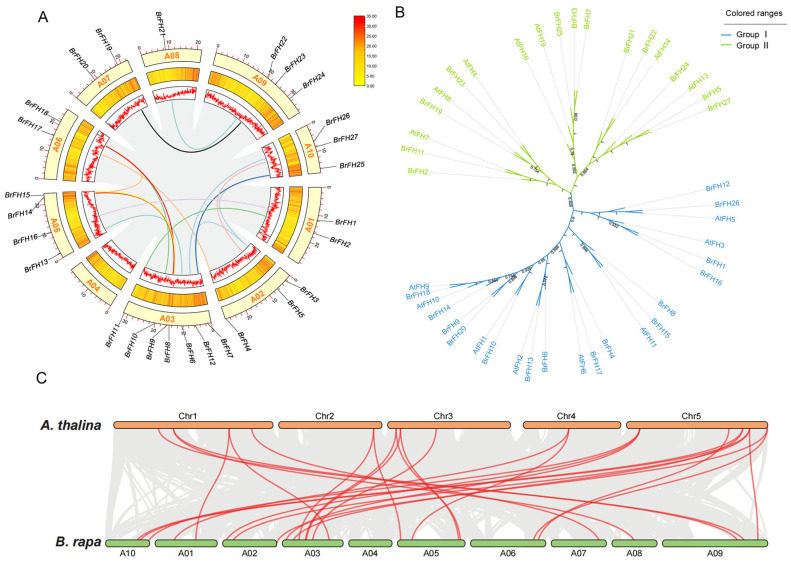
Chromosomal localization, phylogenetic classification and syntenic relationships of *BrFHs* between *B.rapa* and *A. thaliana*. (**A**) *BrFH* gene localization and replication on *B. rapa* chromosomes. The outermost yellow blocks are representative of the 10 chromosomes, and the middle and inner regions show the density of each chromosome. The curves of different colors link the *FH* genes in *B. rapa*, respectively. (**B**) Phylogenetic tree of *FH* in *B. rapa* and *A. thaliana*. Different colors correspond to different groups: blue indicates Group I, green indicates Group II. (**C**) Collinearity analysis between *B. rapa* and *A. thaliana*. Red lines indicate homologous genes.

The chromosomal distribution and synteny of *BrFH* genes were systematically analyzed based on genomic sequence data. A total of 27 *BrFH* genes were mapped onto the nine chromosomes of *B. rapa*, with no *BrFH* gene detected on chromosome 04 (Chr04), indicating an uneven genomic distribution. These *BrFH* genes are discretely distributed across the genome, with each chromosome harboring between one and seven members. Notably, chromosome 03 (Chr03) contains the largest number of *BrFH* genes (seven), whereas chromosome 08 (Chr08) harbors only a single *BrFH* gene (Figure 1A).

To further investigate the evolutionary relationships of *BrFH* genes, synteny analysis was performed between *B. rapa* and *A. thaliana*. A total of 26 *BrFH* genes exhibited syntenic relationships with *FH* genes in *A. thaliana*, indicating a high degree of syntenic conservation and suggesting a close evolutionary relationship between the two species (Figure 1B). To gain deeper insights into the evolutionary relationships within the *BrFH* family, FH proteins from *A. thaliana* and *B. rapa* were used to construct a phylogenetic tree. Phylogenetic analysis classified the BrFH proteins into two distinct groups, designated Group I and Group II (Figure 1C). Group I was the larger clade, comprising 19 *BrFH* members and accounting for 70.4% of the total *BrFH* members, whereas Group II contained eight *BrFH* members. The clustering of FH proteins within the same clades suggests a high degree of homology and implies potential functional similarities among BrFH proteins.

### 3.3. Analysis of Evolutionary Relationship, Gene Structure, and Conservative Motif of FH Family Genes

**Figure 2:**
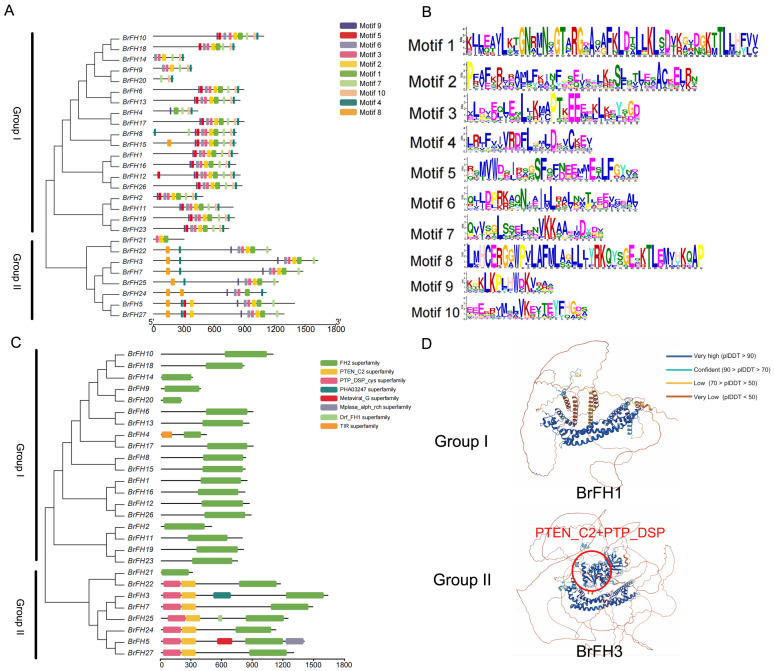
Gene structure and characterization of conserved motifs of *BrFH*s from *B. rapa*. (**A**) The motifs of BrFH proteins. (**B**) The conserved motifs of *BrFHs*. The integral height of each stack indicates the level of conservation at this site, and the amino acid frequency is indicated by the size of each letter. (**C**) The domain regions of BrFHs. (**D**) Protein tertiary structure diagrams showing domain differences in BrFHs between Group I and Group II.

To comprehensively analyze the structural characteristics of *BrFH* genes, we conducted a detailed investigation of motif, conserved domains, and tertiary structure of protein across the 27 members of *BrFH* gene family. Motif analysis of the 27 *BrFH*s revealed the presence of 3 to 10 conserved motifs, with *BrFH20* and *BrFH21* containing the fewest (3 motifs each). Motif 4 was the most conserved, which was present in all genes, except *BrFH21* (Figure 2A,B). Additionally, Motifs 2, 3, and 7 were highly conserved and were present in 92.6% of the genes. In contrast, Motif 8 was the least conserved, and predominantly found in Group II, with 70.3% of all genes containing this motif. Furthermore, Motif 10 was exclusively present in Group I. These findings indicate that the motifs vary among *BrFH* members, especially between the two groups.

All *BrFH* proteins containing a major conserved hallmark domain, FH2 superfamily domain (Figure 2C), which can affect the production or clearance of ROS by regulating the cytoskeleton. For example, the recombination of microfilaments may enhance the localization or activity of antioxidant enzymes such as SOD and CAT, thereby reducing oxidative damage [56]. Interestingly, PTEN-C2 and PTP_DSP_cys superfamily domains were only present in Group II (Figure 2C), which can balance the production and clearance of ROS by regulating the phosphorylation status of signaling pathways [57]. Comparative analysis of the tertiary structures of BrFHs proteins between Group I and Group II revealed distinct structural conservation within each group but significant divergence between the two groups, particularly in the PTEN-C2 and PTP_DSP_cys superfamily domains (Figure 2D and Figure S1). Furthermore, several genes exhibited unique domain compositions, including PHA03247 in *BrFH3*, Metaviral_G and Mplasa_alph_rch in *BrFH5*, TIR in *BrFH4*, and Drf_FH1 in *BrFH25* (Figure 2D). The substantial structural and domain variability among these members suggests potential functional diversification.

### 3.4. Cis-Acting Element Analysis of FH Family Genes

**Figure 3:**
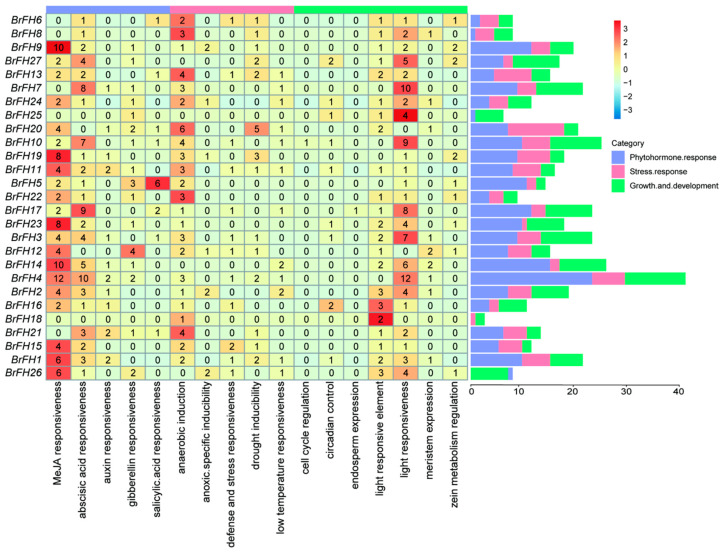
Analysis of *cis*-elements in promoter of *FH* genes in *B. rapa*. Different kinds of *cis*-elements and relative positions in every *BrFH* gene were represented by colored blocks.

To elucidate the potential biological functions of cis-acting regulatory elements within the *BrFH* gene family, we scanned the 2000 bp upstream promoter regions (relative to the transcriptional start site ATG) using the PlantCARE database [58]. The identified cis-regulatory elements were categorized into three major functional groups: (1) growth and development, encompassing elements involved in cell cycle regulation, endosperm-specific expression, light responsiveness, and meristem activity; (2) stress responses, including motifs associated with anaerobic induction, drought inducibility, and low-temperature responsiveness; and (3) phytohormone responses, comprising elements responsive to methyl jasmonate, abscisic acid, auxin, gibberellin, and salicylic acid (Figure 3). Among these genes, *BrFH4* contains the highest number of cis-acting elements, primarily associated with phytohormone responses and plant growth and development, whereas *BrFH18* possesses the fewest, predominantly related to plant growth and development. It is worth noting that approximately 85% of these genes contained abscisic acid, anaerobic induction and light responsiveness elements, indicating that these gene might participate in plant growth and development, and environment stresses in the plant. In addition, *BrFH20* and *BrFH19* may contribute to enhanced tolerance to drought stress, whereas *BrFH2* and *BrFH14* may confer increased tolerance to low-temperature stress by *cis*-acting element analysis. This comprehensive profile indicates that *BrFH* genes are likely modulated by a wide range of physiological processes and environmental cues.

### 3.5. Tissue-Specific Expression Patterns of FH Family Genes

**Figure 4:**
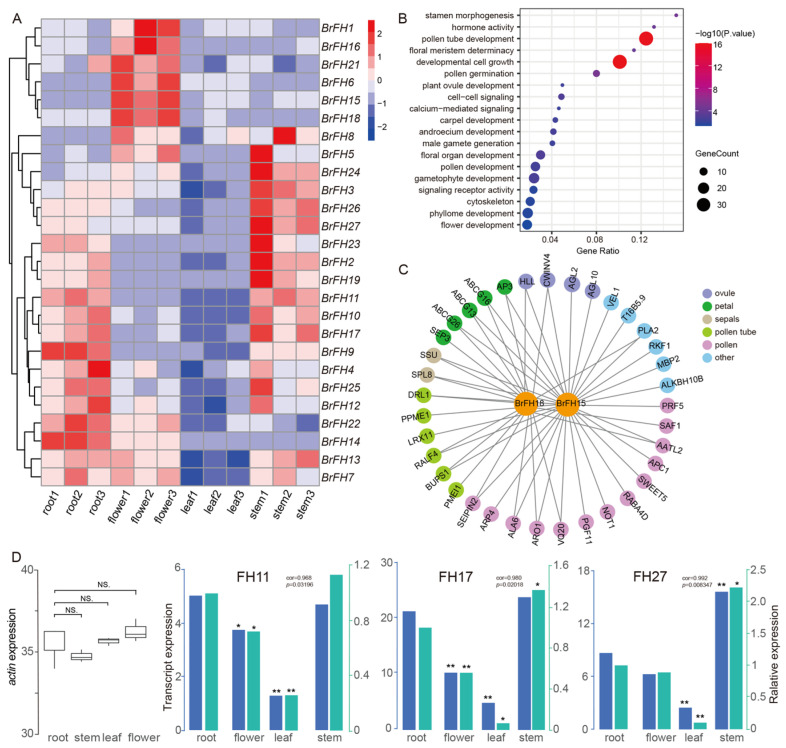
The *BrFH* gene family exhibits differential expression across various tissues. (**A**) Expression profile of *FH* genes in different tissues. (**B**) Gene network co-expression diagram of *BrFH1*, *BrFH15*, *BrFH16* and *BrFH18*. Purple nodes represent *FH* genes, bule nodes represent interacted genes, and the lines connecting the nodes signify interactions between genes. (**C**) Gene enrichment analysis of *BrFH1*, *BrFH15*, *BrFH16* and *BrFH18*. (**D**) Expression analysis of *actin* and three *BrFH* genes selected in roots, flowers, leaves and stems. Data were shown as means. The Pearson correlation coefficients was calculated using R packages (v4.3.3) (N.S., not significant, *t*-test, * *p* < 0.05, ** *p* < 0.01).

We aimed to systematically evaluate across diverse tissues of the expression profiles in the *BrFH* family gene, revealing distinct expression patterns for each gene within the *BrFH* family. The comprehensive analysis encompassed four tissues, including roots, stems, leaves, and flowers (Figure 4A and Table S1). Tissue-specific expression analysis revealed distinct patterns among *BrFH* genes. *BrFH4*, *BrFH9*, *BrFH14*, and *BrFH22* were predominantly expressed in roots, whereas *BrFH2*, *BrFH3*, *BrFH5, BrFH19*, *BrFH23, BrFH24*, *BrFH26*, and *BrFH27* showed high expression levels in stems. In contrast, *BrFH1*, *BrFH6*, *BrFH8*, *BrFH15*, *BrFH16*, *BrFH18,* and *BrFH21* were primarily expressed in flowers and exhibited expression patterns similar to those of floral development-related genes such as *AP3*, *AGL10* and so on (Figure 4B and Table S3). Weighted gene co-expression network analysis (WGCNA) revealed that genes with expression patterns similar to highly flower-expressed *BrFH*s were significantly enriched in functions related to pollen tube development and cell growth processes (Figure 4C). Genes with expression patterns similar to highly stem-expressed *BrFH*s were associated with kinase activity, abscisic acid response, and cell wall biogenesis (Figure S2A,B). Genes with expression patterns resembling those of highly stem- and root-expressed *BrFH*s were potentially regulated by processes such as cell morphogenesis, root development, and transferase activity (Figure S2C,D). In addition, genes with expression patterns similar to highly root-expressed *BrFH*s were enriched in functions related to stimulus response, cell communication, plasma membrane organization, and signal transduction pathways (Figure S2E,F).

To further verify the tissue-specific function of the *FH* family, we chose to quantify the expression levels of three genes (*BrFH11*, *BrFH17*, and *BrFH27)* across different tissues due to its stable expression (Figure 4D). The results showed that *BrFH11*, *BrFH17*, *BrFH23*, and *BrFH27* were predominantly expressed in stems, consistent with the expression profiles obtained from transcriptome data (Figure 4D). Therefore, the above results indicated members of the *BrFH* gene family play an important role in tissue development.

### 3.6. Transcriptome Analysis of BrFHs in Response to Cold Stress

**Figure 5:**
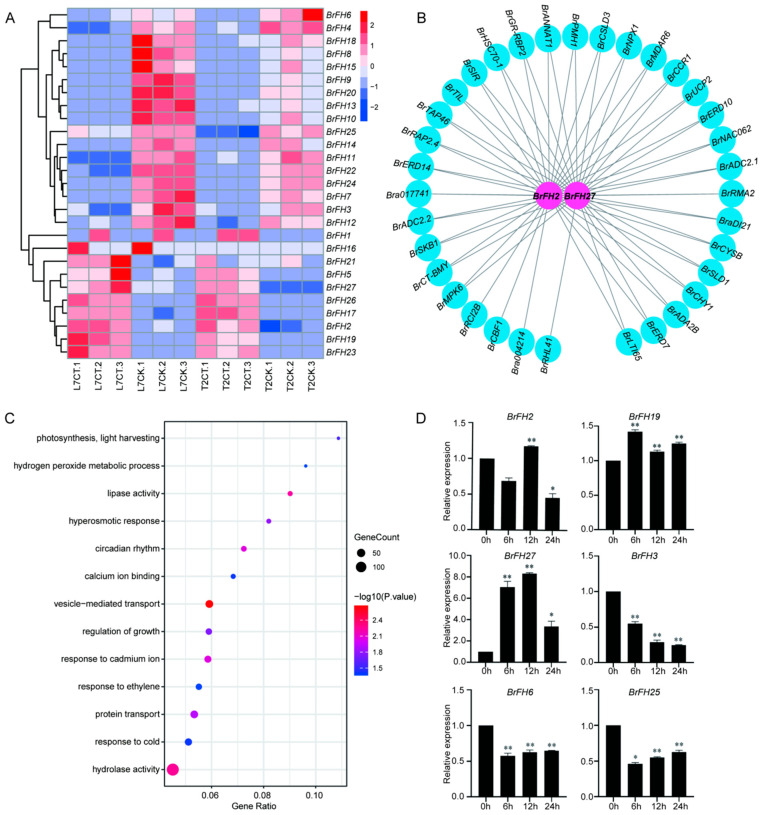
*BrFH* gene family exhibits distinct responses to low-temperature stress. (**A**) The expression of *BrFHs* under 4 °C were detected by RNA-seq. (**B**) Gene network co-expression diagram of *BrFH2* and *BrFH27*. Blue-colored genes represent cold-resistant genes associated with *BrFH2* and *BrFH27*. (**C**) GO enrichment of genes co-expressed with *BrFH2* and *BrFH27* in *B. rapa*. (**D**) Expression analysis of *BrFH* under cold stress at different time treatments. Data were shown as means; errors are shown as ±SD (*t*-test, * *p* < 0.05, ** *p* < 0.01).

To further characterize the *BrFH* gene family in response to low-temperature stress, transcriptome data were retrieved from the NCBI database (Table S1), comprising four treatment groups: L7CK (control under strong cold tolerance), L7CT (cold treatment under strong cold tolerance), T2CK (control under low cold tolerance), and T2CT (cold treatment under low cold tolerance). Among the *BrFH* gene family, *BrFH2*, *BrFH5*, *BrFH17*, *BrFH19*, *BrFH21*, *BrFH23*, *BrFH26*, and *BrFH27* exhibited high expression levels in both strong and low cold-tolerance lines, with higher expression in the strong cold-tolerance line. In contrast, *BrFH3*, *BrFH4*, *BrFH6*, *BrFH7*, *BrFH8*, *BrFH9*, *BrFH10*, *BrFH11*, *BrFH12*, *BrFH13*, *BrFH14*, *BrFH15*, *BrFH18*, *BrFH20*, *BrFH22*, *BrFH24*, and *BrFH25* were down-regulated under cold-tolerance conditions (Figure 5A). Meanwhile, WGCNA revealed that several *BrFH* genes, including *BrFH2*, *BrFH27*, and *BrFH19*, exhibited high expression levels in L7CT and showed expression patterns similar to those of cold-tolerance-related genes such as *CBL1*, *CBF1*, *CCR1* and so on (Figure 5B and Figure S3A–C and Table S4) [59,60]. Genes co-expressed with these *BrFH*s were significantly enriched in biological processes associated with cold responses, hydrolase activity, and vesicle-mediated transport (Figure 5C and Figure S3D), suggesting that these *BrFH*s may contribute to the regulation of cold tolerance mechanisms.

To further validate the expression patterns of these genes under cold stress, we analyzed their transcript levels at 0, 6, 12, and 24 h post-treatment. Among them, *BrFH2*, *BrFH19*, and *BrFH27* exhibited high expression levels, whereas *BrFH3*, *BrFH6*, and *BrFH25* were down-regulated, consistent with the expression patterns observed in the transcriptomic data. Taken together, these findings suggest that *BrFH*s may participate in response to cold stress and have diverse functions.

## 4. Discussion

**Figure 6:**
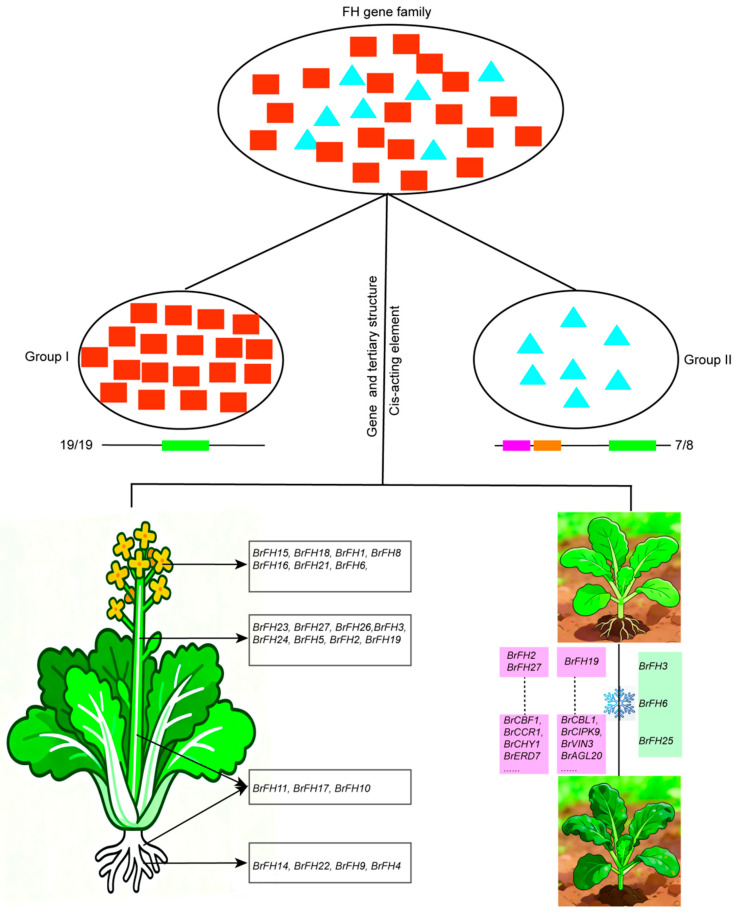
A model illustrating the functional diversification of the *BrFH* gene family. The red frames indicate genes that are up-regulated in response to cold stress. The green frames indicate genes that are down-regulated in response to cold stress.

*B. rapa* is an economically vegetable that includes a wide range of leafy vegetables and oilseed crops cultivated worldwide [61]. As an important vegetable, understanding the molecular mechanisms underlying growth, development, and stress adaptation in *B. rapa* is of both theoretical and practical significance. Formins are key regulators of the actin cytoskeleton in eukaryotic cells, functioning as actin nucleation factors that initiate microfilament polymerization and contribute to the organization and dynamic remodeling of actin essential for plant growth, development, and responses to abiotic stress [62]. To date, the formin gene family has been reported in several plant species, including *A. thaliana* and rice [15,16], but their functions in *B. rapa* remain unexplored. In this study, we identified and analyzed the physicochemical properties, protein structures, and expression patterns of 27 *BrFH*s during tissue development and in response to cold stresses (Figure 6). These results enhance our understanding of the roles of *BrFH*s in the molecular mechanisms underlying tissue development and cold stress responses.

Formins are proteins defined by the existence of FH-domain-containing, representing an evolutionarily conserved protein family. Evolutionary analyses revealed that the *FH* gene family is present in Chlorophytina plants, undergoing a progressive increase in gene copy number during plant evolution (Figure S4A and Table S5). This is particularly evident in magnoliids, monocots, and eudicots, suggesting lineage-specific amplification of the gene family throughout angiosperm evolution. The *FH* gene family was divided into two distinct subfamilies, consistent with the classification of *FH* genes reported in many other plant species [32,63]. Members of this family are characterized by two evolutionarily conserved and functionally divergent domains. Comparative domain analysis revealed that the FH2 domain is universally present in all examined plant lineages, whereas PTP_PTEN-like and PTP_DSP_cys superfamily appear to have emerged later during evolution and were first detected in Bryophyta (Figure S4B), suggesting lineage-specific domain acquisition during early land plant evolution. Meanwhile, the abundance of PTP_PTEN-like and PTP_DSP_cys superfamily domains increased markedly in eudicots, indicating that these newly acquired domains may have undergone expansion and functional diversification, thereby contributing more extensively to the biological functions of FH proteins in eudicot lineages. In addition, the *BrFH* gene family is relatively conserved within the species and across the examined plant taxa (Figure S4C). Furthermore, *FH* genes in plant lineages that evolved after Chlorophytina appear to have been predominantly subjected to purifying selection (Figure S4D), while exhibiting greater functional diversification in monocots and eudicots, indicating that these genes have maintained essential core functions while undergoing lineage-specific specialization during angiosperm evolution.

Gene structure and *cis*-regulatory elements play critical roles in shaping gene function by modulating transcriptional regulation, expression patterns, and functional specificity [64,65]. Here, motif and domain analyses revealed that the *BrFH* gene family can be divided into two distinct groups, consistent with the observed phylogenetic relationships, suggesting that evolutionary divergence is accompanied by structural differentiation (Figure 6). In addition, *BrFH*s harbor diverse cis-regulatory elements, which likely modulate their transcriptional responses to developmental cues and environmental stimuli, thereby contributing to the functional diversification.

Formins are proteins characterized by an FH2 domain and the ability to nucleate linear F-actin de novo, playing a pivotal role in cytoskeletal regulation and tissue development [2,66]. *BrFH*s exhibit distinct tissue-specific expression patterns (Figure 6). Genes with profiles similar to *BrFH15* and *BrFH18*, which are highly expressed in flowers, were enriched for functions associated with floral organ development, particularly pollen tube growth, including key genes such as *AP3*, *SEP*3, and *LRX11*. Genes co-expressed with *BrFH2* and *BrFH27*, highly expressed in stems, were enriched in kinase activity, calmodulin binding, and cell–cell communication. Similarly, genes with expression patterns resembling *BrFH9*, highly expressed in roots, were enriched in processes related to root morphogenesis and cell communication. The tissue-specific high expression of *BrFH*s indicates their contribution to tissue development and functional specialization, likely reflecting their roles in regulating cytoskeletal dynamics in different organs.

*B. rapa* possesses a robust capacity for cold acclimation, which confers significant chilling and freezing tolerance, thereby enabling the plant to successfully propagate, survive overwintering periods, and establish itself in temperate environments [67]. The actin cytoskeleton encoded by *FH* genes undergoes remodeling and interacts with various membranes (plasma membrane, vacuolar membrane, and nuclear membrane) under low-temperature stress, drought stress, and other types of stress in response to damage from the external environment. We found that *BrFH2*, *BrFH27*, and *BrFH9* are highly expressed in cold-tolerant *B. rapa* plants under cold stress (Figure 6). Notably, *BrFH2* and *BrFH27* exhibited expression patterns similar to those of *CBF1*, *CCR1*, *CHY1*, *ERD7*, and other cold-responsive genes. Furthermore, two cold-responsive cis-regulatory elements were identified in the promoter region of *BrFH2*, suggesting that these elements may regulate its transcriptional activity and contribute to enhanced cold tolerance in *B. rapa*. In contrast, *BrFH9* shows a co-expression pattern with genes such as *CBL1*, *CIPK9*, *VIN3*, and *AGL20*. We hypothesize that *BrFH2*, *BrFH27*, and *BrFH9* may induce cytoskeletal change under cold stress, thereby facilitating Ca^2+^ influx and activating *CBF*–*COR* and *CBL1*–*CIPK9* signaling pathways, ultimately enhancing cold tolerance [68,69,70]. Upon sensing calcium signaling changes, CBL interacts with and activates CIPK9, which subsequently modulates processes including ion transport and gene expression through phosphorylation of downstream target proteins, thereby contributing to the plant’s cold-resistance response. These results suggest that *BrFH2*, *BrFH27*, and *BrFH9* may play important roles in the cold stress response of *B. rapa*, potentially by coordinating with key cold-responsive pathways. Specifically, *BrFH2* and *BrFH27* may function in conjunction with the *CBF*-mediated transcriptional network, while *BrFH9* may act through *CBL*-*CIPK* signaling to regulate downstream gene expression and ion homeostasis, highlighting the diverse mechanisms by which *BrFH*s contribute to cold tolerance.

## 5. Conclusions

In conclusion, this study systematically identified and characterized the formin gene family in *B. rapa*. The analyses of gene structure, phylogeny, cis-regulatory elements, and expression patterns indicate that *BrFH*s have diversified functions in tissue development and responses to abiotic stress, particularly cold stress. These findings provide a foundation for future functional studies of *BrFH*s and contribute to a better understanding of actin cytoskeleton regulation in *B. rapa*.

## Figures and Tables

**Table 1:** Information of *FH* family members in *B. rapa*.

Gene ID	Gene Name	Chr. No	Gene Start Position	Gene End Position	*A.thaliana* ID	*A. thaliana* Name	Subcellular Localization	Amino Acid (aa)	CDS (bp)	MW (kDa)	Isoelectric Point
*Bra039561*	*BrFH1*	A01	11,886,057	11,889,150	*AT5G54650*	*AtFH5*	Plasma membrane	837	2514	92.22	9.44
*Bra035415*	*BrFH2*	A01	16,477,720	16,479,274	*AT1G59910*	*AtFH7*	Plasma membrane	490	1473	53.40	7.07
*Bra028684*	*BrFH3*	A02	2,564,374	2,571,652	*AT5G07770*	*AtFH16*	Nucleus	1627	4884	175.30	7.39
*Bra031838*	*BrFH4*	A02	27,611,349	27,613,482	*AT5G67470*	*AtFH6*	Cytoplasm	440	1323	49.71	6.02
*Bra020385*	*BrFH5*	A02	6,491,606	6,497,627	*AT5G58160*	*AtFH13*	Chloroplast	1396	4191	155.28	8.30
*Bra000328*	*BrFH6*	A03	10,561,399	10,564,306	*AT2G43800*	*AtFH2*	Plasma membrane	895	2688	98.20	8.47
*Bra005952*	*BrFH7*	A03	1,373,493	1,380,081	*AT5G07770*	*AtFH16*	Nucleus	1480	4443	160.67	6.85
*Bra001148*	*BrFH8*	A03	14,976,355	14,979,096	*AT3G05470*	*AtFH11*	Plasma membrane	823	2472	91.02	8.82
*Bra001256*	*BrFH9*	A03	15,545,258	15,546,720	*AT3G07540*	*AtFH10*	Cytoplasm	381	1146	42.77	5.30
*Bra013217*	*BrFH10*	A03	19,804,375	19,807,889	*AT3G25500*	*AtFH1*	Plasma membrane	1091	3276	119.27	8.14
*Bra017889*	*BrFH11*	A03	31,345,005	31,356,742	*AT1G59910*	*AtFH7*	Plasma membrane	790	2373	85.99	9.03
*Bra029012*	*BrFH12*	A03	5,866,076	5,869,007	*AT5G54650*	*AtFH5*	Plasma membrane	859	2580	93.57	8.36
*Bra004786*	*BrFH13*	A05	1,882,215	1,825,011	*AT2G43800*	*AtFH2*	Chloroplast	857	2574	94.10	8.64
*Bra029655*	*BrFH14*	A05	22,786,695	22,787,755	*AT3G07540*	*AtFH10*	Chloroplast	303	912	34.09	4.87
*Bra039436*	*BrFH15*	A05	23,324,560	23,327,524	*AT3G05470*	*AtFH11*	Plasma membrane	819	2460	91.19	8.86
*Bra037087*	*BrFH16*	A05	9,934,103	9,936,953	*AT5G54650*	*AtFH5*	Plasma membrane	816	2451	89.67	9.11
*Bra024447*	*BrFH17*	A06	15,802,206	15,805,576	*AT5G67470*	*AtFH6*	Plasma membrane	897	2694	98.69	9.25
*Bra037491*	*BrFH18*	A06	21,200,697	21,203,353	*AT5G48360*	*AtFH2*	Chloroplast	807	2424	88.31	5.78
*Bra016233*	*BrFH19*	A07	18,713,686	18,716,094	*AT1G70140*	*AtFH8*	Chloroplast, Vacuole	802	2409	87.64	9.51
*Bra012246*	*BrFH20*	A07	9,097,123	9,097,833	*AT3G07540*	*AtFH10*	Cytoplasm	196	591	22.01	4.75
*Bra038438*	*BrFH21*	A08	8,735,316	8,737,759	*AT1G31810*	*AtFH14*	Cytoplasm	303	912	34.14	8.73
*Bra023204*	*BrFH22*	A09	20,717,185	20,722,757	*AT1G31810*	*AtFH14*	Nucleus	1163	3492	128.06	6.72
*Bra024640*	*BrFH23*	A09	23,866,999	23,869,323	*AT1G24150*	*AtFH4*	Vacuole	744	2235	81.94	9.20
*Bra007822*	*BrFH24*	A09	31,070,385	31,075,379	*AT2G25050*	*AtFH18*	Cytoplasm	1118	3357	124.06	8.37
*Bra009306*	*BrFH25*	A10	16,073,796	16,079,751	*AT5G07770*	*AtFH16*	Chloroplast	1237	3714	137.59	5.86
*Bra002969*	*BrFH26*	A10	6,365,088	6,368,134	*AT5G54650*	*AtFH5*	Plasma membrane	877	2634	95.18	9.09
*Bra002668*	*BrFH27*	A10	8,324,945	8,330,743	*AT5G58160*	*AtFH13*	Cytoplasm	1292	3879	142.04	6.67
Average	\	\	\	\	\	\	\	849.33	2548	93.33	7.68

## Data Availability

The original contributions presented in this study are included in the article/Supplementary Material. Further inquiries can be directed to the corresponding author.

## Supplementary Materials

The following supporting information can be downloaded at: https://www.mdpi.com/article/10.3390/genes17020207/s1, Figure S1: Tertiary structure analysis of FHs in *B. rapa*. Different colors correspond to different groups: (A) Group I, (B) Group II. (C) Proteins of BrFHs with shorter polypeptide chains. Figure S2: (A–F) Gene co-expression network construction and Gene Ontology (GO) enrichment analysis of genes sharing similar expression patterns with BrFHs. Figure S3: Weighted gene co-expression network analysis. (A) Co-expression network module of *BrFH19*. (B) Transcriptomic expression analysis of *BrFH19*. (C) Gene network diagram of *BrFH19*. (D) Gene enrichment analysis of *BrFH19*. Figure S4: Phylogenetic relationships of plant species and distribution of FH proteins. (A) Phylogenetic relationships among plant species from Chlorophytina to eudicots, and the distribution of BrFH proteins across selected species analyzed in this study. The green block represents the FH domain. The magenta and orange represent PTEN-C2 and PTP_DSP_cys superfamily. (B) Number of FH proteins. The green blocks represent the number of genes containing only the FH domain, whereas the red blocks represent the number of genes containing the FH domain together with PTEN-C2 and PTP_DSP_cys superfamily domains. (C) Comparative analysis of Ka values across plant species from chlorophytina to eudicots. (D) Comparative analysis of Ka/Ks values across plant species from chlorophytina to eudicots. Table S1: The source of transcriptome data under different tissues and Cold treatments. Table S2: List of primers used for qRT-PCR. Table S3: The co-expression of *BrFH15* and *BrFH18* in flowers. Table S4: The co-expression of *BrFH2*, *BrFH27* and *BrFH19* in response to cold stress. Table S5: The classification and genomic data source of eight species.
