# Supplementary material for: Comprehensive Analysis of Formin Genes Reveals Their Roles in Tissue Development and Cold Stress Responses in Brassica rapa
Source: Genes (Basel). 2026 Feb 9;17(2):207. doi: 10.3390/genes17020207 (PMC12941170; doi:10.3390/genes17020207)
Supplement: Supplementary file 1 [file genes-17-00207-s001.zip › Supplementary Figures.pdf]

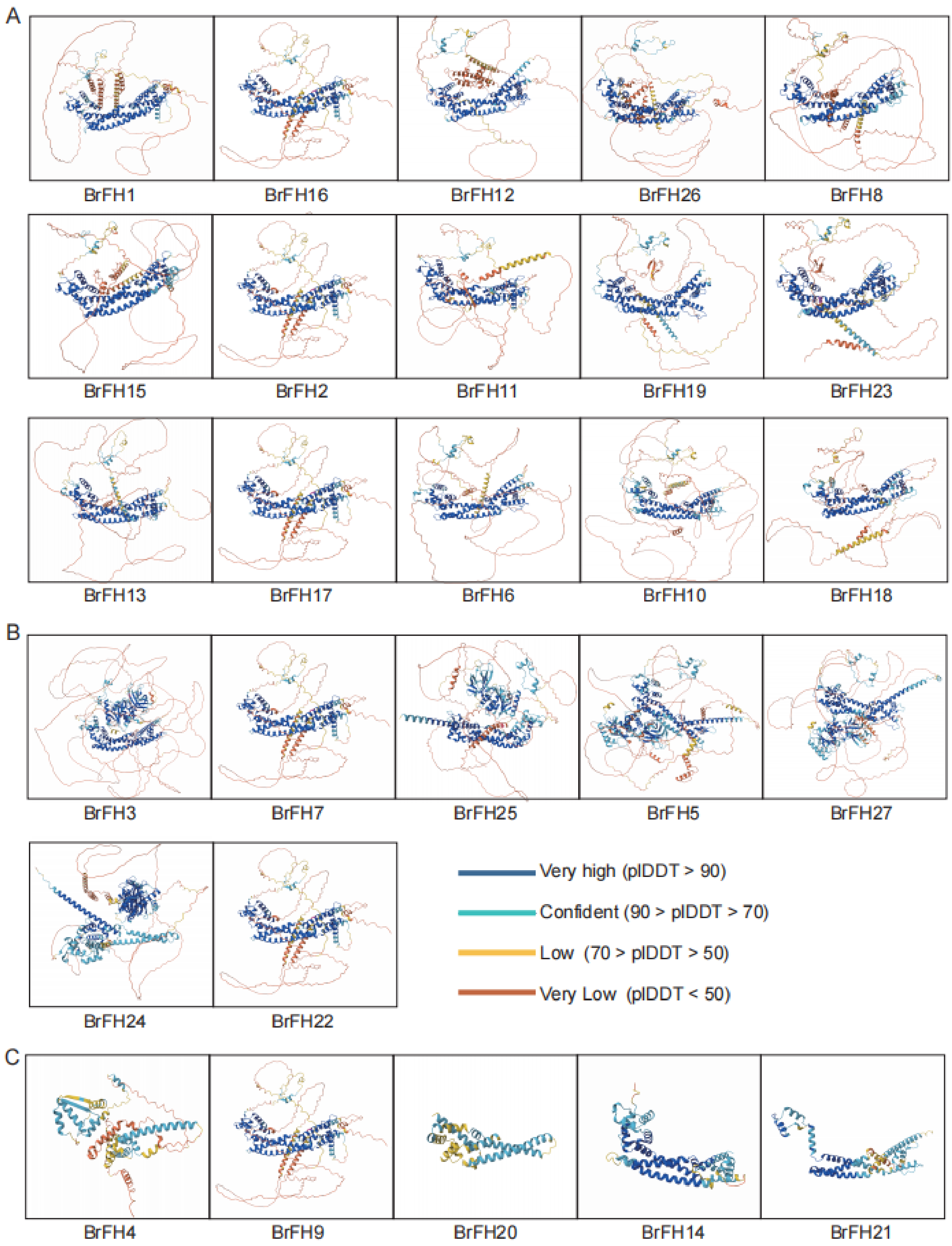

Figure S1. Tertiary structure analysis of FHs in *B. rapa*. Different colors correspond to different groups: (**A**)Group I, (**B**) Group II. (**C**) Proteins of BrFHs with shorter polypeptide chains.

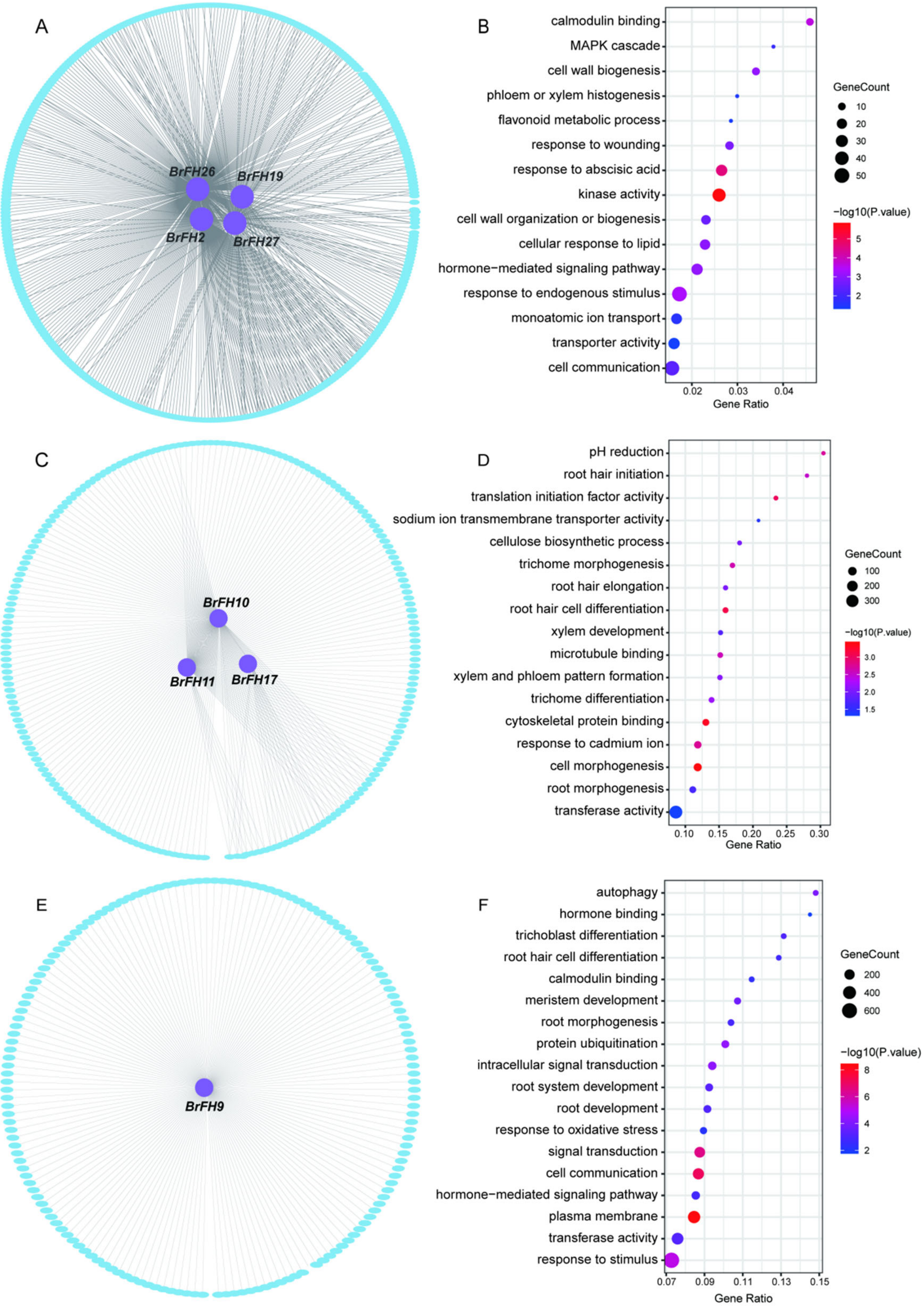

Figure S2. (**A-F**) Gene co-expression network construction and Gene Ontology (GO) enrichment analysis of genes sharing similar expression patterns with *BrFH*s.

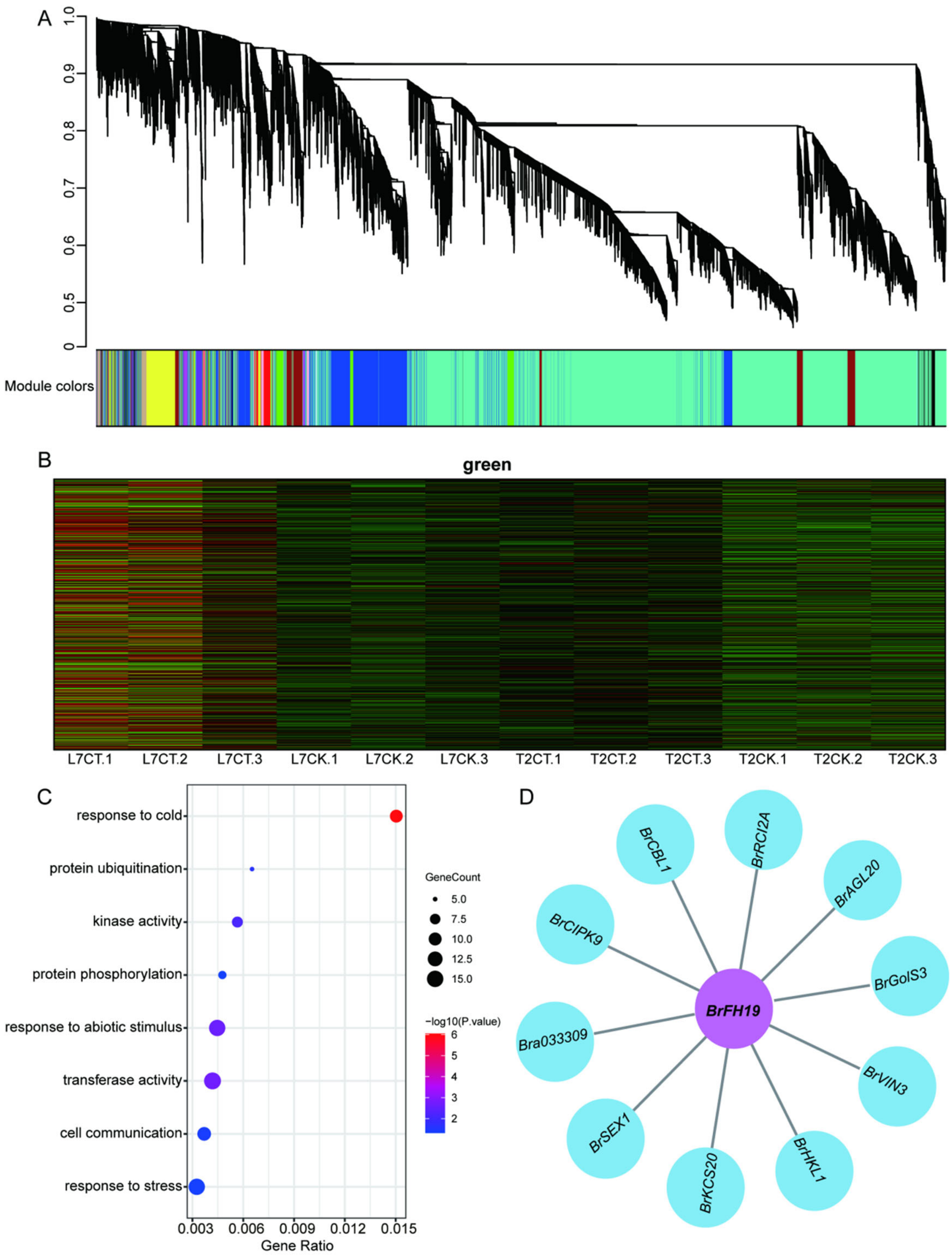

Figure S3. **Weighted gene co-expression network analysis.** (**A**) Co-expression network module of *BrFH19*. (**B**) Transcriptomic expression analysis of *BrFH19*. (**C**) Gene network diagram of *BrFH19*. (**D**) Gene enrichment analysis of *BrFH19*.

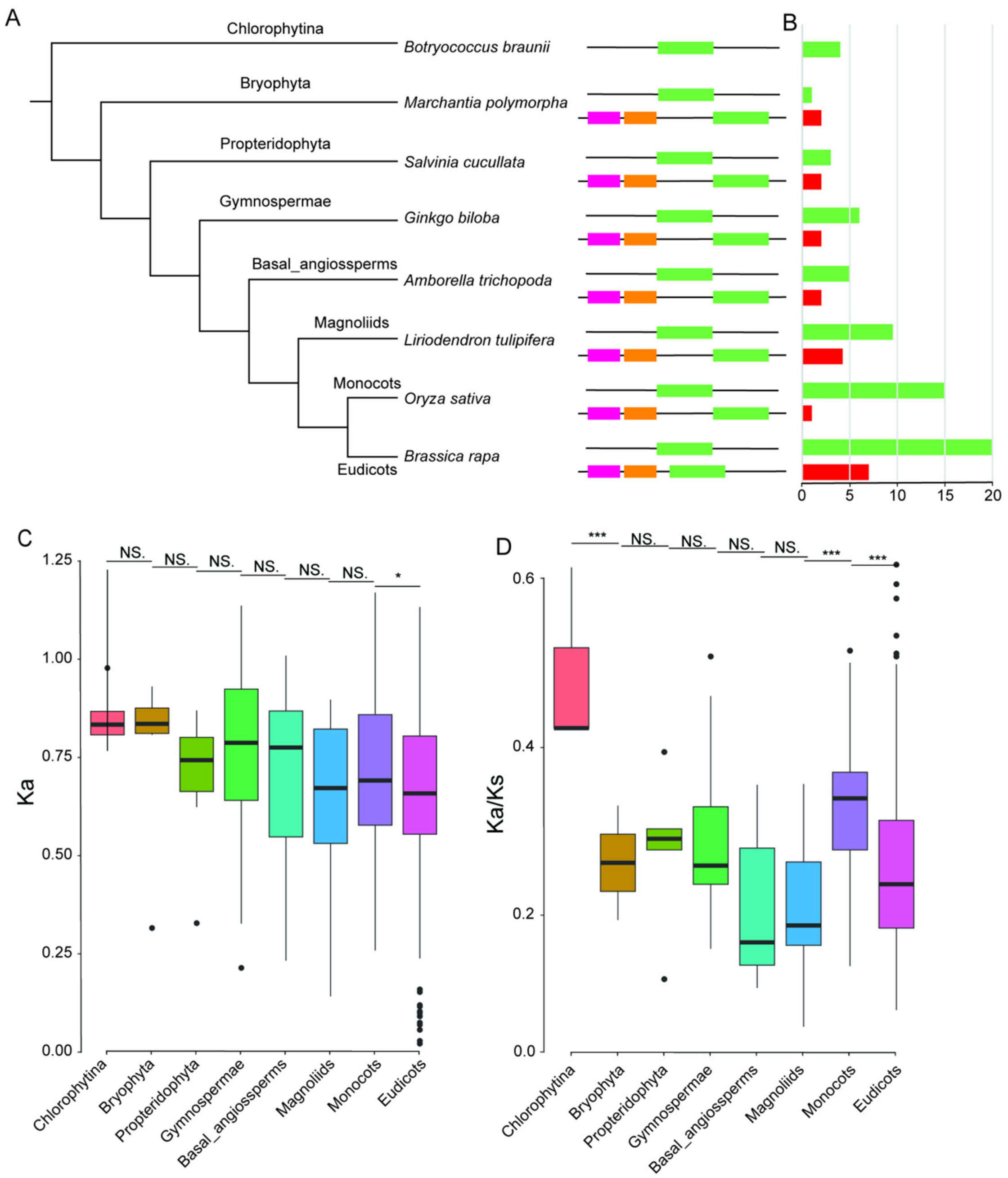

Figure S4. Phylogenetic relationships of plant species and distribution of FH proteins. (A) Phylogenetic relationships among plant species from Chlorophytina to eudicots, and the distribution of BrFH proteins across selected species analyzed in this study. The green block represent FH doamin. The magenta and orange represent PTEN-C2 and PTP_DSP_cys superfamily. (B) Number of FH proteins. The green blocks represent the number of genes containing only the FH domain, whereas the red blocks represent the number of genes containing the FH domain together with PTEN-C2 and PTP_DSP_cys superfamily domains. (C) Comparative analysis of *K*a values across plant species from chlorophytina to eudicots. D. Comparative analysis of *K*a/*K*s values across plant species from chlorophytina to eudicots.
